# WHO Global Survey on Maternal and Perinatal Health in Latin America: classifying caesarean sections

**DOI:** 10.1186/1742-4755-6-18

**Published:** 2009-10-29

**Authors:** Ana P Betrán, A Metin Gulmezoglu, Michael Robson, Mario Merialdi, João P Souza, Daniel Wojdyla, Mariana Widmer, Guillermo Carroli, Maria R Torloni, Ana Langer, Alberto Narváez, Alejandro Velasco, Anibal Faúndes, Arnaldo Acosta, Eliette Valladares, Mariana Romero, Nelly Zavaleta, Sofia Reynoso, Vicente Bataglia

**Affiliations:** 1 Department of Reproductive Health and Research, World Health Organization, Geneva, Switzerland; 2 National Maternity Hospital, Dublin, Ireland; 3 Centro Rosarino de Estudios Perinatales, Rosario, Argentina; 4 Department of Obstetric and Gynecology, Federal University of Sao Paulo, Sao Paulo, Brazil; 5 EngenderHealth, New York, USA; 6 Fundación Salud, Ambiente y Desarrollo, Quito, Ecuador; 7 Hospital Docente Ginecobstétrico "America Arias", La Habana, Cuba; 8 Centro de Pesquisas em Saúde Reprodutiva de Campinas (CEMICAMP), Campinas, Brazil; 9 Department of Obstetrics and Gynecology, Universidad Nacional de Asunción, Asunción, Paraguay; 10 Universidad Nacional Autónoma de Nicaragua, León, Nicaragua; 11 Centro de Estudios de Estado y Sociedad, Buenos Aires, Argentina; 12 Instituto de Investigación Nutricional, Lima, Peru; 13 The Population Council, Latin America Office, Mexico City, Mexico; 14 Department of Obstetrics and Gynecology, Hospital Nacional de Itaguá, Asunción, Paraguay

## Abstract

**Background:**

Caesarean section rates continue to increase worldwide with uncertain medical consequences. Auditing and analysing caesarean section rates and other perinatal outcomes in a reliable and continuous manner is critical for understanding reasons caesarean section changes over time.

**Methods:**

We analyzed data on 97,095 women delivering in 120 facilities in 8 countries, collected as part of the 2004-2005 Global Survey on Maternal and Perinatal Health in Latin America. The objective of this analysis was to test if the "10-group" or "Robson" classification could help identify which groups of women are contributing most to the high caesarean section rates in Latin America, and if it could provide information useful for health care providers in monitoring and planning effective actions to reduce these rates.

**Results:**

The overall rate of caesarean section was 35.4%. Women with single cephalic pregnancy at term without previous caesarean section who entered into labour spontaneously (groups 1 and 3) represented 60% of the total obstetric population. Although women with a term singleton cephalic pregnancy with a previous caesarean section (group 5) represented only 11.4% of the obstetric population, this group was the largest contributor to the overall caesarean section rate (26.7% of all the caesarean sections). The second and third largest contributors to the overall caesarean section rate were nulliparous women with single cephalic pregnancy at term either in spontaneous labour (group 1) or induced or delivered by caesarean section before labour (group 2), which were responsible for 18.3% and 15.3% of all caesarean deliveries, respectively.

**Conclusion:**

The 10-group classification could be easily applied to a multicountry dataset without problems of inconsistencies or misclassification. Specific groups of women were clearly identified as the main contributors to the overall caesarean section rate. This classification could help health care providers to plan practical and effective actions targeting specific groups of women to improve maternal and perinatal care.

## Background

Caesarean section (CS) rates have increased significantly worldwide during the last decades but in particular in middle and high income countries [1-3]. In several countries of Latin America, the proportion of deliveries by CS is approaching 40% at national level [1,3]. In United States, the CS rate in 2006 was 31.1% [4], and the latest estimates for several European countries are also above 30% [1]. This steady increase has fuelled the debate over acceptable rates of CS and the risk-benefit analysis in ensuring optimum maternal and perinatal outcomes in different populations with different access to health resources. The medical consequences of a rising CS rate remain uncertain and the implications in developing countries may be more significant because of the impact it could have on limited resources [2,3,5,6].

Auditing of CS rates is carried out in many countries. However there is no standardized, internationally accepted method for classifying CS and thereby enabling the assessment of both maternal and neonatal outcomes in clinically relevant groups of women. Most studies on CS rates have used indications as a method of analysis and while this does provide interesting information on why the CS took place, it does not enable completion of the audit cycle mainly because the information cannot be used to change care prospectively.

In 2001, a new classification for CS known as the "10-group" or "Robson classification" was described [7]. This classification provides a framework for monitoring, auditing and analysing CS rates at facility level in an action-oriented manner, and it can be applied consistently with minimal resources. This classification is based on four obstetric concepts (Table 1) and classifies women in 10 groups (Table 2). These groups are mutually exclusive, totally inclusive, clinically relevant and prospectively identifiable. The characteristics of this classification system allow the reporting and analysis of data in a clinically meaningful manner in relevant groups of women. It allows comparisons over time in one unit and between different units, providing practical grounds to change practice in specific prospective groups of women. While the 10 groups are standard for initial and robust comparative purposes, each group can be subdivided further or some groups can be amalgamated in order to adapt to the needs of different settings. Indications for CS can be applied within the different groups.

**Table 1:** Obstetric concepts and variables used to classify women in the 10-group or Robson classification.

Obstetric concept	Variable
Category of pregnancy	Single cephalic pregnancy
	Single breech pregnancy
	Single oblique or transverse lie
	Multiple pregnancies
	
Previous obstetric history	Nulliparous
	Multiparous without uterine scar
	Multiparous with uterine scar
	
Course of pregnancy	Spontaneous labour
	Induced labour
	Caesarean section before labour
	
Gestation	Gestational age in completed weeks at time of delivery

**Table 2:** Obstetric characteristics of women included in each of the 10 groups.

Group	Women included
1	Nulliparous with single cephalic pregnancy, ≥37 wks gestation in spontaneous labour

2*	Nulliparous with single cephalic pregnancy, ≥37 wks gestation who either had labour induced or were delivered by CS before labour

3	Multiparous without a previous uterine scar, with single cephalic pregnancy, ≥37 wks gestation in spontaneous labour

4*	Multiparous without a previous uterine scar, with single cephalic pregnancy, ≥37 wks gestation who either had labour induced or were delivered by CS before labour

5	All multiparous with at least one previous uterine scar, with single cephalic pregnancy, ≥37 wks gestation

6	All nulliparous women with a single breech pregnancy

7	All multiparous women with a single breech pregnancy including women with previous uterine scars

8	All women with multiple pregnancies including women with previous uterine scars

9	All women with a single pregnancy with a transverse or oblique lie, including women with previous uterine scars

10	All women with a single cephalic pregnancy ≤36 wks gestation, including women with previous scars

* Often divided into 2a and 4a (inductions) and 2b and 4b (pre-labour CS)

In 2004, WHO initiated the Global Survey on Maternal and Perinatal Health project. The main objectives of this survey were to develop a network of health institutions worldwide to assess how evidence-based recommendations are implemented in maternal and perinatal health care, to identify gaps at the facility and sub-national levels, and to assist in effective planning, implementation and monitoring [8]. WHO envisions to keep this network of health facilities active, in order to intermittently collect and analyse data on priority research questions, in a real-time framework.

In this context, we set out to perform a secondary analysis of the 2004-2005 WHO Global Survey in Latin America [9,10] using the 10-group classification. Our objectives were twofold. Firstly, to see if the classification could be successfully applied to a large dataset. Secondly, to identify the groups of women that contribute most to the high rates of caesarean deliveries in Latin America and test how this classification can be used to identify problems and challenges and subsequently enable actions to be taken. Since the type of facility (tertiary/referral vs. other) and the educational level of the mother play an important role on CS rates [10], this analysis includes assessment by type of facility and level of education of the women.

## Methods

The WHO Global Survey was implemented in Latin America in 2004-2005. The focus of this survey was to explore the relation between rate of caesarean delivery and maternal and perinatal outcomes. The detailed methodology of the WHO global survey has been described elsewhere [8-10]. Briefly, this was a facility-based study of women delivering in randomly selected health facilities in 23 geographical areas in eight randomly selected Latin American countries (i.e. Argentina, Brazil, Cuba, Ecuador, Mexico, Nicaragua, Paraguay, and Peru). A stratified multistage cluster sampling design was used to obtain a sample of health institutions [11]. Individual informed consent was not sought (except for Brazil) as data were collected at the institutional level from medical records without identifying the individual women. The ethics committee of each participating institution and the Scientific and Ethical Review Group of the UNDP/UNFPA/WHO/World Bank Special Programme of Research, Development and Research Training in Human Reproduction, Geneva, Switzerland approved the study.

The study population consisted of all women admitted for delivery over a 3-month period in institutions with up to 6,000 deliveries per year, and over a 2-month period for those institutions with more than 6,000. In each health institution, data pertaining to the characteristics and services available, and individual-woman data were collected. Information related to each woman was extracted from the medical records by trained data collectors within a day after delivery and for the period that the women were in the hospital. Information collected included demographic characteristics, maternal risk indicators, mode of delivery, and maternal and newborn outcomes up to hospital discharge [8-10].

All necessary information to implement the 10-group classification (see Table 1) was collected through the survey without prior knowledge of the 10-group classification. Data were processed using SAS System (version 9.1.3). In this manuscript we present an overall analysis of the 10-group classification as well as of the individual countries. We wanted to test whether the combination of certain obstetric characteristics as defined by the 10-group classification (e.g. parity, presentation, gestational age, type of labour initiation and previous mode of delivery) would be associated with selected social factors (e.g. educational level). We therefore analysed the risk of CS in each group according to the educational level of the mother and the type of facility (tertiary/referral vs. other) as crude and adjusted odds ratio (OR) with 95% confidence intervals. The variables considered in the models were those used in previous published analysis of this survey [9,10].

## Results

In Latin America, the 2004-2005 WHO Global Survey included 120 institutions in eight countries. For the stipulated period of time and according to hospital records, 106,546 deliveries occurred in these institutions. Information was collected for 97,095 deliveries giving a 91% coverage of the survey. The contribution of each institution to the total number of deliveries ranged from 37 to 4536. Thirty-five out of the 120 institutions contributed with over 1,000 deliveries.

Table 3 represents the overall 10-group classification table. It includes, for each of the 10 groups and overall, the number of deliveries, the number of CS, and the proportion of deliveries by CS. The highest and lowest country values are shown in parenthesis. From these numbers the relative size of each group, and the absolute and relative contribution of each group to the overall CS rate can be calculated. The overall rate of CS was 35.4%, which means that approximately one in three women delivered by CS during the study period. Rates varied from 30.8% in Nicaragua to 40.3% in Ecuador.

**Table 3:** Standard 10-group (Robson) classification table, 2004-2005 Global Survey in Latin America.

Group (a)	Obstetric population (b)	Relative size of the group (n, % and range) (c)	CS rate (n, % and range) (d)	Absolute contribution to CS rate (% and range) (e)	Relative contribution to CS rate (% and range) (f)
1	Nulliparous with single cephalic pregnancy, ≥37 wks gestation in spontaneous labour	26576 27.7 (23.1-32.2)	6172 23.2 (13.9-37.2)	6.4 (4.0-10.0)	18.2 (13.5-24.8)
2	Nulliparous with single cephalic pregnancy, ≥37 wks gestation who either had labour induced or were delivered by CS before labour	8376 8.7 (5.6-14.3)	5142 61.4 (41.7-74.0)	5.4 (2.8-8.4)	15.2 (6.9-23.6)
3	Multiparous without a previous uterine scar, with single cephalic pregnancy, ≥37 wks gestation in spontaneous labour	30909 32.3 (26.4-34.8)	3044 9.9 (4.5-17.3)	3.2 (1.5-6.0)	9.0 (4.3-14.9)
4	Multiparous without a previous uterine scar, with single cephalic pregnancy, ≥37 wks gestation who either had labour induced or were delivered by CS before labour	6704 7.0 (3.8-10.5)	2822 42.1 (22.8-60.4)	3.0 (1.5-5.3)	8.3 (3.7-14.9)
5	All multiparous with at least one previous uterine scar, with single cephalic pregnancy, ≥37 wks gestation	10890 11.4 (8.7-14.1)	9042 83.0 (76.9-95.9)	9.4 (7.9-12.0)	26.7 (23.6-32.2)
6	All nulliparous women with a single breech pregnancy	1409 1.5 (1.0-2.0)	1258 89.3 (82.2-91.0)	1.3 (0.9-1.8)	3.7 (2.2-5.3)
7	All multiparous women with a single breech pregnancy including women with previous uterine scars	1794 1.9 (1.5-2.7)	1482 82.6 (77.8-88.1)	1.6 (1.2-2.2)	4.4 (3.7-6.2)
8	All women with multiple pregnancies including women with previous uterine scars	954 1.0 (0.6-1.3)	690 72.3 (63.6-82.0)	0.7 (0.4-1.0)	2.0 (1.0-3.0)
9	All women with a single pregnancy with a transverse or oblique lie, including women with previous uterine scars	1419 1.5 (0.4-3.8)	1335 94.1 (77.6-100.0)	1.4 (0.3-3.6)	3.9 (1.0-8.9)
10	All women with a single cephalic pregnancy ≥36 wks gestation, including women with previous scars	6773 7.1 (4.7-9.2)	2913 43.0 (32.8-50.5)	3.0 (1.5-4.3)	8.6 (4.2-11.4)
	Total	95804 100	33900 35.4	35.4	100

Standard 10-group classification table including: (a) group number in the 10-group classification; (b) description of the obstetric population in of each group; (c) number and proportion of the obstetric population in each group; (d) number and proportion of CS in each group; (e) absolute contribution of each group to the total CS rate; (f) relative contribution of each group to the total CS rate. Highest and lowest country proportions are presented in parenthesis.

**Figure 1:**
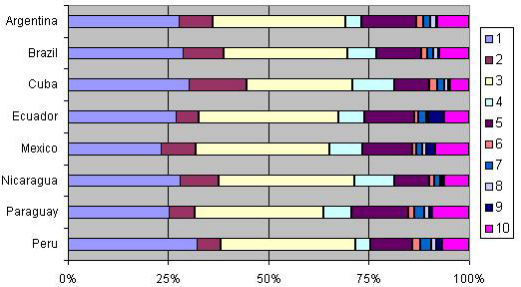
**Obstetric population by Robson group**.

**Figure 2:**
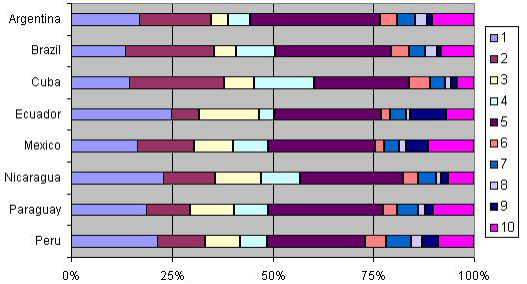
**Relative contribution to the caesarean section rate by Robson group**.

Groups 1 and 3 (women with single cephalic pregnancy, ≥37 weeks gestation without previous CS who entered into labour spontaneously) are the largest groups representing 60% of the obstetric population included in this analysis (see Table 3 and Fig 1). The third largest is group 5 (women with single cephalic pregnancy, ≥37 weeks gestation who have already undergone at least one CS), which represents 11.4% of the obstetric population. CS rates in each of these groups are 23.2% (group 1), 9.9% (group 3), and 83% (group 5), respectively. However, the largest contributions to the total CS rate are groups 1, 2 and 5 which were responsible for 21.2% of the 35.4% overall CS rate in this survey (Table 3 and Fig 2).

Groups 2 (nulliparous) and 4 (multiparous) with single cephalic pregnancy, ≥37 weeks gestation without previous CS who either had labour induced or were delivered by CS before labour started, present high rates of CS (61.4% and 42.1%, respectively) and represent about 16% of the women in the survey (see Table 3). Groups 6 through 10 present high rates of CS due to the particular obstetric conditions within which these are defined. However, their contribution to the overall CS rate is smaller (8% out of 35.4%) due to the relatively small size of these groups. Among these groups, the larger contributor to the overall CS is group 10 which includes all women with single cephalic pregnancy at ≤ 36 weeks gestation.

The rates for the individual countries are shown in Additional file 1 and they can be analysed according to the principles described above. Each country's 10 groups can be compared with the overall population for size of the groups, CS rates within the groups and the contribution of each group. Fig 1 and 2 show country-level data for the 10-group classification. Fig 1 presents per country how the obstetric population is distributed in each of the groups (proportion of women in each group) while Fig 2 shows the relative contributions of each group to the total CS rate (the contribution of each group depends on the size as well as the CS rate in the group). The population in the largest groups (1 and 3) varied from 23% in Mexico to 32% in Peru (group 1) and from 26.4% in Cuba to 34.8% in Ecuador (group 3). On the other hand the contribution of the different groups to the total CS rates presented more variability. For nulliparous in group 1, the relative contributions ranged from about 14% in Brazil to 25% in Ecuador. The contribution to the total CS rate by nulliparous in group 2 ranged from 7% in Ecuador to 24% in Cuba (see Fig 2).

The stratified analysis according to type and complexity of institution and maternal education did not show any statistically significant differences. Adjusted OR of having a CS in each of the 10 groups did not show statistically significant differences according to education level of the mother or the type of facility (tertiary/referral vs. other). That is, education and type of facility do not seem to be risk factors for CS in any of the groups (Additional file 2).

## Discussion

The 10-group classification system for CS was easily applied in the large WHO Global Survey dataset which involved 97,095 deliveries representing one of the largest dataset on maternal and perinatal health for the region. Furthermore, because most of the deliveries in these countries occur in health facilities, these results are believed to represent current practices during childbirth in the region. The necessary variables for the classification were readily available and are well defined which minimizes inconsistencies even when data come from different hospitals and countries and even if the data are not primarily collected for the purpose of the generation of these statistics. This suggests its usefulness from a public health perspective given that data collection in a consistent manner is a constant challenge.

Analysis using the 10-group classification identified groups of women in whom relatively high or low rates of caesarean delivery could be expected. By classifying women in this way, subgroups requiring closer monitoring can be identified for more in-depth analyses.

For example, group 3, multiparous women with a singleton fetus in a normal cephalic presentation, who have not had a CS before and who enter labour spontaneously at term, usually constitutes the largest group among all delivering women representing, in this survey, 32.3% of the obstetric population. Compared with other groups, these women are less likely to have obstetric indications for CS since they present very low risk in general. Hence, the CS rate in this group can be expected to be low. If a rise in CS rate is observed in this group, it could indicate that CS is being performed without a medical reason or that women are being misclassified with regard to their history of caesarean delivery. In fact, group 3 is normally so low risk and such a standard management is usually applied that it could be used to assess the quality of the data collection regarding this classification. Nevertheless, the almost 10% CS rate in this group is rather high compared with 1% in the National Maternity Hospital in Dublin in 2006 [12], or 3.7% in the Royal Women's Hospital in Melbourne in 2005 [13].

The second largest group among delivering women was found to be nulliparous women with a singleton fetus in the normal cephalic position entering labour spontaneously at term; group 1 (27.7% of the women). In this group too, women are less likely to have medical indications for CS, but it may be required for complications of labour such as dystocia or fetal distress. The CS rate in this group can be expected to be relatively low and it is a key indicator of the CS rate in the same women in future pregnancies. However, the 23.2% of CS in group 1, contrast markedly with other published rates such as the 6.7% in the National Maternity Hospital in Dublin in 2006 [12], or 14.8% in New Jersey in 2004 [14].

Group 5, women with a previous CS and a single fetus in normal cephalic presentation at term constituted the next important group in the Latin American dataset (11.4% of the delivering women). Additionally, this group made the highest contribution to the overall CS rate (about 27% of all CS; see Table 3). In a context of overall increase of CS rates, it is critical to consider this group seriously because as CS rates increase in the other groups, group 5 will increase its size and therefore it will become an even more important contributor to the overall CS rate. However, reducing CS in this group is likely to be most difficult because having a previous delivery by CS increases the likelihood of caesarean delivery in the next pregnancy. The important message must be to try and prevent the first CS.

Groups 2 and 4 come next in terms of size, 8.7% and 7%, respectively. Nulliparous and multiparous without previous CS, respectively, who have a singleton fetus in cephalic presentation at term, who have labour induced or delivered by CS before labour constituted the next largest groups in the Global Survey dataset. Rates were 61.4% and 42.1% in group 2 and 4, respectively. These high CS rates indicate that a considerable proportion of women either had a high incidence of conditions that required labour induction (such as pre-eclampsia at term) or had elective labour inductions and pre-labour CS for the sake of convenience or other potentially non-medical reason. Clearly, these groups would need to be investigated in more detail to understand the exact reasons of the high rates and take appropriate action. By reviewing the indications for ending the pregnancy before spontaneous labour (i.e. by CS before labour and labour induction) and how labour induction was managed in these women, one could identify gaps in the application of evidence-based clinical practices and potentially reduce unnecessary CS in these groups.

Owing to their obstetric factors such as multiple pregnancies, breech presentation, transverse or oblique lie, women in the groups 6-10 can be expected to have higher CS rates. However, the contributions of these groups to the overall CS rate would be low, considering the size of this population. One further point in relation to group 9 is that by definition this group should have a caesarean section rate of 100% and therefore, it is also a group that can be used to assess the quality of data collection.

The present analysis did not include further stratification of each of the 10 groups of women. However, this would be advisable in countries or institutions attempting to understand practices in certain obstetrics groups and their related levels of CS. The classification presents the flexibility to allow for this stratification. Particularly, groups 2, 4, and 5 can benefit from subdivision into those women who had labour induced and those who were delivered by pre-labour CS. Another useful subdivision would be in group 5, where women with previous uterine scars could be subdivided into those with only one previous CS, and women with two or more previous CS. Additionally, the study of outcomes and characteristics of women with multiple CS could also provide evidence and assist to understand potential adverse effects of CS in these women, a group that could be possibly growing in developing countries. Furthermore, this classification can embed the indications for CS classification in the sense that indications can be applied within the different groups. Other in-depth analysis could also stratify women in each group by other risk factors or medical conditions, age, race, BMI, case mix, or evening/day shifts, among others [15,16].

Although the 10-group classification has been used in different units in countries worldwide [13,14,17-19], this is the first time it was tested using data from a large multicountry study providing an auditing framework and grounds for comparisons between facilities and countries in the same study. It proved to be a practical and easy way of identifying the main groups of women who most contribute to the overall rate of CS. Since this classification is based upon well defined parameters (see Tables 1 and 2), inconsistencies in classification are very unlikely. Being able to compare CS rates in a reliable and consistent manner over time and between units and countries is one of the persisting challenges at the moment which this classification will be capable to overcome. In this respect and in order to compare the 10-group classification with other available systems, we would recommend that future research and steps include a systematic review of CS classification systems published and analyze the advantages and deficiencies of each system.

## Conclusion

This classification identified specific groups of women as the main contributors to the overall CS rate in Latin America; groups that could be targeted for relevant effective actions. Result from this analysis should encourage local investigators and health authorities to use data on CS rates in innovative approaches in order to maximize the use of the collected information, disseminate the results and think of strategies to reduce these rates when appropriate.

## Authors' contributions

*Acquisition of primary data and survey implementation*: GC (regional coordinator), AL, AN, AV, AF, AA, EV, MR, NZ, SR, VB (country coordinators).

*Secondary analysis idea and design of analysis plan*: APB, MG, MR, MM, DW.

*Analysis and interpretation of the data*: APB, MG, MR, MM, DW.

*Drafting of the manuscript*: APB, MM, JPS.

*Critical revision of the manuscript for important intellectual content*: APB, MG, MR, MM, JPS, DW, MW, MRT.

All authors read and approved the final manuscript

## Supplementary Material

Additional file 1 **Data by country**. This file presents the tables for the 10-group classification for each of the 8 countries in this survey. Click here for file

Additional file 2 **Stratified analysis**. This file presents the results from the stratified analysis by type of institution and education of the mother. Click here for file
