# Supplementary material for: WHO Global Survey on Maternal and Perinatal Health in Latin America: classifying caesarean sections
Source: Reprod Health. 2009 Oct 29;6:18. doi: 10.1186/1742-4755-6-18 (PMC2779175; doi:10.1186/1742-4755-6-18)
Supplement: Additional file 1 — Data by country. This file presents the tables for the 10-group classification for each of the 8 countries in this survey. [file 1742-4755-6-18-S1.doc]

**Additional file 1**

**Data by country**

Table 1: Standard 10-group classification table by country: number of CS and number of deliveries by group, proportion of the obstetrical population in each group, proportion of CS in each group, contribution of each group to the total CS rate. The 2004-1005 Global Survey in 8 countries.

|  | **ARGENTINA** | | | |  | **BRAZIL** | | | |
| --- | --- | --- | --- | --- | --- | --- | --- | --- | --- |
| **Group** | **Relative size (no. and %)** | **CS (No. and %)** | **Contribution to CS (%)** | **Relative contribution (%)** |  | **Relative size (no. and %)** | **CS (No. and %)** | **Contribution to CS (%)** | **Relative contribution (%)** |
| 1 | 2881  27.7 | 611  21.2 | 5.9 | 16.8 |  | 4142  28.6 | 575  13.9 | 4.0 | 13.5 |
| 2 | 886  8.5 | 656  74.0 | 6.3 | 17.9 |  | 1471  10.2 | 934  63.5 | 6.5 | 22.0 |
| 3 | 3435  33.0 | 156  4.5 | 1.5 | 4.3 |  | 4475  30.9 | 234  5.2 | 1.6 | 5.4 |
| 4 | 422  4.1 | 199  47.2 | 1.9 | 5.4 |  | 1041  7.2 | 426  40.9 | 2.9 | 9.8 |
| 5 | 1411  13.6 | 176  83.4 | 11.3 | 32.2 |  | 1598  11.1 | 1229  76.9 | 8.5 | 28.7 |
| 6 | 179  1.7 | 158  88.3 | 1.5 | 4.3 |  | 218  1.5 | 191  87.6 | 1.3 | 4.4 |
| 7 | 197  1.9 | 172  87.3 | 1.6 | 4.6 |  | 217  1.5 | 170  78.3 | 1.2 | 4.1 |
| 8 | 134  1.3 | 102  76.1 | 1.0 | 2.8 |  | 178  1.2 | 132  74.2 | 0.9 | 3.0 |
| 9 | 45  0.4 | 39  86.7 | 0.4 | 1.1 |  | 58  0.4 | 45  77.6 | 0.3 | 1.0 |
| 10 | 818  7.9 | 383  46.8 | 3.7 | 10.5 |  | 1064  7.4 | 349  32.8 | 2.4 | 8.1 |
| Total | 10408  100.0 | 3652  35.1 | 35.1 |  |  | 14462  100.0 | 4285 | 29.6 |  |

|  | **CUBA** | | | |  | **ECUADOR** | | | |
| --- | --- | --- | --- | --- | --- | --- | --- | --- | --- |
| **Group** | **Relative size (no. and %)** | **CS (No. and %)** | **Contribution to CS (%)** | **Relative contribution (%)** |  | **Relative size (no. and %)** | **CS (No. and %)** | **Contribution to CS (%)** | **Relative contribution (%)** |
| 1 | 3817  30.2 | 640  16.8 | 5.1 | 14.3 |  | 3330  26.9 | 1239  37.2 | 10.0 | 24.8 |
| 2 | 1813  14.3 | 1059  58.4 | 8.4 | 23.6 |  | 693  5.6 | 344  49.6 | 2.8 | 6.9 |
| 3 | 3341  26.4 | 337  10.1 | 2.7 | 7.6 |  | 4314  34.8 | 744  17.3 | 6.0 | 14.9 |
| 4 | 1331  10.5 | 671  50.4 | 5.3 | 14.9 |  | 829  6.7 | 189  22.8 | 1.5 | 3.7 |
| 5 | 1105  8.7 | 1060  95.9 | 8.4 | 23.6 |  | 1519  12.3 | 1324  87.2 | 10.7 | 26.6 |
| 6 | 247  2.0 | 222  89.9 | 1.8 | 5.1 |  | 129  1.0 | 111  86.1 | 0.9 | 2.2 |
| 7 | 198  1.6 | 165  83.3 | 1.3 | 3.7 |  | 243  2.0 | 201  82.7 | 1.6 | 4.0 |
| 8 | 121  1.0 | 77  63.6 | 0.6 | 1.7 |  | 69  0.6 | 47  68.1 | 0.4 | 1.0 |
| 9 | 77  0.6 | 71  92.2 | 0.5 | 1.4 |  | 472  3.8 | 451  95.6 | 3.6 | 8.9 |
| 10 | 592  4.7 | 194  32.8 | 1.5 | 4.2 |  | 779  6.3 | 340  43.7 | 2.8 | 6.9 |
| Total | 12642  100.0 | 4496  35.6 |  |  |  | 12377  100.0 | 4990  40.3 |  |  |

|  | **MEXICO** | | | |  | **NICARAGUA** | | | |
| --- | --- | --- | --- | --- | --- | --- | --- | --- | --- |
| **Group** | **Relative size (no. and %)** | **CS (No. and %)** | **Contribution to CS (%)** | **Relative contribution (%)** |  | **Relative size (no. and %)** | **CS (No. and %)** | **Contribution to CS (%)** | **Relative contribution (%)** |
| 1 | 4827  23.1 | 1302  27.0 | 6.2 | 16.4 |  | 1574  27.9 | 393  25.0 | 7.0 | 22.7 |
| 2 | 1826  8.7 | 1103  60.4 | 5.3 | 14.0 |  | 540  9.6 | 225  41.7 | 4.0 | 13.0 |
| 3 | 6958  33.4 | 761  10.9 | 3.7 | 9.8 |  | 1903  33.8 | 200  10.5 | 3.5 | 11.4 |
| 4 | 1681  8.1 | 684  40.7 | 3.3 | 8.7 |  | 562  10.0 | 169  30.1 | 3.0 | 9.7 |
| 5 | 2611  12.5 | 2087  79.9 | 10.0 | 26.5 |  | 488  8.7 | 443  90.8 | 7.9 | 25.6 |
| 6 | 205  1.0 | 180  87.8 | 0.9 | 2.4 |  | 67  1.2 | 61  91.0 | 1.1 | 3.6 |
| 7 | 335  1.6 | 291  86.9 | 1.4 | 3.7 |  | 92  1.6 | 76  82.6 | 1.4 | 4.5 |
| 8 | 161  0.8 | 132  82.0 | 0.6 | 1.6 |  | 37  0.6 | 25  67.6 | 0.4 | 1.3 |
| 9 | 477  2.3 | 441  92.5 | 2.1 | 5.6 |  | 29  0.5 | 29  100.0 | 0.5 | 1.6 |
| 10 | 1785  8.5 | 902  50.5 | 4.3 | 11.4 |  | 344  6.1 | 115  33.4 | 2.0 | 6.5 |
| Total | 20866  100.0 | 7883  37.8 |  |  |  | 5636  100.0 | 1736  30.8 |  |  |

|  | **PARAGUAY** | | | |  | **PERU** | | | |
| --- | --- | --- | --- | --- | --- | --- | --- | --- | --- |
| **Group** | **Relative size (no. and %)** | **CS (No. and %)** | **Contribution to CS (%)** | **Relative contribution (%)** |  | **Relative size (no. and %)** | **CS (No. and %)** | **Contribution to CS (%)** | **Relative contribution (%)** |
| 1 | 859  25.0 | 269  31.3 | 7.8 | 18.6 |  | 5146  32.2 | 1143  22.2 | 7.2 | 21.2 |
| 2 | 226  6.6 | 157  69.5 | 4.6 | 11.0 |  | 921  5.8 | 664  72.1 | 4.1 | 12.1 |
| 3 | 1105  32.2 | 156  14.1 | 4.6 | 11.0 |  | 5378  33.6 | 456  8.5 | 2.9 | 8.6 |
| 4 | 237  6.9 | 121  51.1 | 3.5 | 8.3 |  | 601  3.8 | 363  60.4 | 2.3 | 6.8 |
| 5 | 484  14.1 | 412  85.1 | 12.0 | 28.6 |  | 1674  10.5 | 1311  78.3 | 8.2 | 24.2 |
| 6 | 55  1.6 | 50  90.9 | 1.5 | 3.6 |  | 309  1.9 | 285  82.2 | 1.8 | 5.3 |
| 7 | 84  2.5 | 74  88.1 | 2.2 | 5.2 |  | 428  2.7 | 333  77.8 | 2.1 | 6.2 |
| 8 | 37  1.1 | 24  64.9 | 0.7 | 1.7 |  | 217  1.3 | 151  69.6 | 0.9 | 2.7 |
| 9 | 28  0.8 | 27  96.4 | 0.8 | 1.9 |  | 233  1.5 | 232  99.6 | 1.4 | 4.1 |
| 10 | 315  9.2 | 149  47.3 | 4.3 | 10.2 |  | 1076  6.7 | 481  44.7 | 3.0 | 8.8 |
| Total | 3430  100.0 | 1439  42.0 |  |  |  | 15983  100.0 | 5419  33.9 |  |  |
