# Supplementary material for: WHO Global Survey on Maternal and Perinatal Health in Latin America: classifying caesarean sections
Source: Reprod Health. 2009 Oct 29;6:18. doi: 10.1186/1742-4755-6-18 (PMC2779175; doi:10.1186/1742-4755-6-18)
Supplement: Additional file 2 — Stratified analysis. This file presents the results from the stratified analysis by type of institution and education of the mother. [file 1742-4755-6-18-S2.doc]

**Additional file 2**

**Stratified analysis**

Table 1: Caesarean section rates by group and type of institution (tertiary, secondary and primary).

| **Group** | **Tertiary /Referral** | | **Primary/ Secondary** | | **Tertiary/Referral vs Primary/Secondary** | | **Adjusted*** | |
| --- | --- | --- | --- | --- | --- | --- | --- | --- |
|  | **N** | **%** | **N** | **%** | **OR** | **95% CI** | **OR** | **95% CI** |
| 1 | 3968 | 26.6 | 2200 | 18.9 | 1.55 | 1.46 - 1.65 | 1.79 | 0.77 - 4.16 |
| 2 | 2760 | 64.2 | 2377 | 59.3 | 1.23 | 1.13 - 1.35 | 1.96 | 0.93 - 1.42 |
| 3 | 1894 | 11.2 | 1149 | 8.3 | 1.39 | 1.29 - 1.50 | 1.76 | 0.69 - 4.46 |
| 4 | 1420 | 44.5 | 1396 | 41.2 | 1.14 | 1.04 - 1.26 | 1.80 | 0.82 - 3.94 |
| 5 | 5551 | 82.7 | 3477 | 83.7 | 0.93 | 0.84 - 1.03 | 1.31 | 0.46 - 3.74 |
| 6 | 799 | 89.1 | 458 | 89.6 | 0.94 | 0.66 - 1.34 | 1.86 | 0.40 - 8.73 |
| 7 | 944 | 81.9 | 536 | 83.9 | 0.87 | 0.67 - 1.13 | 1.24 | 0.35 - 4.34 |
| 8 | 469 | 73.9 | 219 | 69.5 | 1.24 | 0.92 - 1.67 | 1.78 | 0.71 - 4.46 |
| 9 | 805 | 94.9 | 436 | 92.2 | 1.59 | 1.01 - 2.50 | 2.24 | 0.33 - 15.20 |
| 10 | 2134 | 46.7 | 776 | 35.5 | 1.59 | 1.43 - 1.77 | 2.08 | 0.99 - 4.38 |

*Adjusted by Anaesthesia during Labour, Maternal Age, Marital Status and Vaginal Bleeding During 1st Half of Pregnancy.

Table 2: Caesarean section rates by level of education of the mother.

| **Group** | **<7 years** | | **≥7 years** | | **<7 years vs ≥7 years** | | **Adjusted*** | |
| --- | --- | --- | --- | --- | --- | --- | --- | --- |
|  | **N** | **%** | **N** | **%** | **OR** | **95% CI** | **OR** | **95% CI** |
| 1 | 1219 | 23.8 | 4650 | 22.9 | 1.05 | 0.98 - 1.13 | 1.09 | 0.96 - 1.24 |
| 2 | 766 | 54.7 | 3985 | 62.1 | 0.73 | 0.66 - 0.83 | 0.79 | 0.50 - 1.25 |
| 3 | 921 | 9.5 | 2054 | 10.2 | 0.93 | 0.85 - 1.01 | 1.00 | 0.91 - 1.11 |
| 4 | 656 | 36.2 | 2074 | 45.5 | 0.68 | 0.61 - 0.76 | 0.75 | 0.51 - 1.11 |
| 5 | 2079 | 80.2 | 6344 | 83.6 | 0.80 | 0.71 - 0.89 | 0.82 | 0.62 - 1.07 |
| 6 | 203 | 88.7 | 968 | 89.4 | 0.93 | 0.59 - 1.46 | 1.10 | 0.42 - 2.88 |
| 7 | 387 | 79.6 | 1030 | 83.9 | 0.75 | 0.57 - 0.98 | 0.80 | 0.50 - 1.27 |
| 8 | 162 | 68.4 | 466 | 72.9 | 0.80 | 0.58 - 1.11 | 0.77 | 0.51 - 1.15 |
| 9 | 358 | 94.5 | 946 | 94.4 | 1.01 | 0.60 - 1.70 | 1.46 | 0.48 - 4.44 |
| 10 | 676 | 38.7 | 2062 | 44.1 | 0.80 | 0.71 - 0.89 | 0.94 | 0.83 - 1.07 |

*Adjusted by Maternal Age, Marital Status, Parity, Previous Stillbirth/Neonatal Death, Caesarean Section in Previous Delivery, Hypertensive Disorders during Pregnancy, Vaginal Bleeding During 1st Half Pregnancy, Condyloma Acuminatta and Referral Status.
